# Supplementary material for: Influence of Paratuberculosis Vaccination on the Local Immune Response in Experimentally Infected Calves: An Immunohistochemical Analysis
Source: Animals (Basel). 2025 Jun 22;15(13):1841. doi: 10.3390/ani15131841 (PMC12248800; doi:10.3390/ani15131841)
Supplement: Supplementary file 1 [file animals-15-01841-s001.zip › animals-3644924-supplementary.pdf]

Table S1. List of primary antibodies used for IHC and antigen retrieval specifications. [a]PTLink (Dako-Agilent® technologies, Santa Clara, USA).

Target	Manufacturer	Reference	Clonality	Antigen Retrieval[a]	Dilution Titer
**TLR1**	GeneTex®	GTX47794	Polyclonal	pH 6.0	1:200
**TLR2**	Invitrogen®	MA1-40080	Monoclonal mT2.4	pH 6.0	1:100
**TLR4**	Biorbyt®	ORB11489	Polyclonal	pH 9.0	1:800
**TLR9**	GeneTex®	GTX31295	Polyclonal	pH 9.0	1:1000
**IFN-γ**	Serotec®	MCA2112	Monoclonal CC330	pH 9.0	1:300
**iNOS**	Invitrogen®	MA3-030	Monoclonal NOS-3F7-B11 B5	pH 6.0	1:300
**CD204**	TransGenic Inc.®	KAL-KT022	Monoclonal SRA-E5	pH 6.0	1:400
**MAP**	Custom Antibody	N/A	Polyclonal	N/D	1:5000

Table S2. Results of the *post-hoc* Tukey's Honestly Significant Difference test for all pairwise comparisons in the model classified with ΔAICC < 2 for the number of TLR1, TLR2, TLR4, IFN-y immunolabeled cells in the intestine according to the infection (IS) and vaccination status (VS).

Marker	Linear hypotheses	Estimate	SE	t-ratio	*p* value
	IS (IF) — IS (NIF) = 0	2.423	0.049	9.433	<0.001
**TLR1**	VS (V) — VS (NV) = 0	1.752	0.321	7.082	0.982
	IS (IF)*VS (V) — IS (IF)*VS (NV) = 0	0.330	0.044	3.449	1.000
	IS (NIF)*VS (V) — IS (NIF)*VS (NV) = 0	0.399	0.040	2.082	0.991
	IS (IF) — IS (NIF) = 0	0.877	0.619	4.160	<0.001
**TLR2**	VS (V) — VS (NV) = 0	1.221	0.421	7.911	0.851
	IS (IF)*VS (V) — IS (IF)*VS (NV) = 0	0.410	0.061	4.669	0.951
	IS (NIF)*VS (V) — IS (NIF)*VS (NV) = 0	0.783	0.045	6.272	0.981
	IS (IF) — IS (NIF) = 0	0.329	0.047	2.046	<0.001
**TLR4**	VS (V) — VS (NV) = 0	0.851	0.042	5.765	0.721
	IS (IF)*VS (V) — IS (IF)*VS (NV) = 0	0.111	0.043	2.260	0.991
	IS (NIF)*VS (V) — IS (NIF)*VS (NV) = 0	0.434	0.038	5.439	0.298
	IS (IF) — IS (NIF) = 0	0.692	0.352	5.628	<0.001
**IFN-y**	VS (V) — VS (NV) = 0	0.762	0.293	6.577	0.892
	IS (IF)*VS (V) — IS (IF)*VS (NV) = 0	0.521	0.051	6.022	0.769
	IS (NIF)*VS (V) — IS (NIF)*VS (NV) = 0	0.557	0.050	4.079	0.940

SE, standard error., IF, infected., NIF, non-infected., V, vaccinated., NV, non-vaccinated.,* interaction.

Table S3. Results of the *post-hoc* Tukey's Honestly Significant Difference test for all pairwise comparisons in the model classified with ΔAICC < 2 for the number of TLR1, TLR2, TLR4, TLR9, IFN-y, iNOS and CD204 immunolabeled cells according to the type of lesion (TL) associated with paratuberculosis infection and vaccination status (VS).

**Marker**	**Linear hypotheses**	**Estimate**	**SE**	**t-ratio**	***p* value**
	VS (V)*TL (C) — VS(V)*TL (F) = 0	-2.892	0.923	-10.291	<0.001
	VS (V)*TL (C) — VS(V)*TL (MF) = 0	-3.298	1.982	-12.771	<0.001
	VS (V)*TL (F) — VS(V)*TL (MF) = 0	0.188	0.013	2.412	0.228
	VS (NV)*TL (C) — VS(NV)*TL (F) = 0	-0.069	0.012	-0.812	0.043
**TLR1**	VS (NV)*TL (C) — VS(NV)*TL (MF) = 0	-0.153	0.039	-1.098	<0.001
	VS (NV)*TL (F) — VS(NV)*TL (MF) = 0	-0.189	0.087	-1.087	0.095
	VS (NV)*TL (C) — VS(NV)*TL (DP) = 0	0.174	0.050	1.776	<0.001
	VS (NV)*TL (F) — VS(NV)*TL (DP) = 0	-2.762	0.872	-5.866	<0.001
	VS (NV)*TL (MF) — VS(NV)*TL (DP) = 0	-1.666	0.451	-4.222	<0.001
	VS (V)*TL (C) — VS(V)*TL (F) = 0	-0.311	0.044	-0.122	0.772
	VS (V)*TL (C) — VS(V)*TL (MF) = 0	-0.122	0.093	-0.091	0.119
	VS (V)*TL (F) — VS(V)*TL (MF) = 0	1.231	0.221	2.811	0.012
	VS (NV)*TL (C) — VS(NV)*TL (F) = 0	-0.021	0.012	-0.240	0.888
**TLR2**	VS (NV)*TL (C) — VS(NV)*TL (MF) = 0	-0.112	0.054	-0.980	0.329
	VS (NV)*TL (F) — VS(NV)*TL (MF) = 0	-0.221	0.101	-2.112	0.100
	VS (NV)*TL (C) — VS(NV)*TL (DP) = 0	-2.552	0.221	-9.323	<0.001
	VS (NV)*TL (F) — VS(NV)*TL (DP) = 0	-1.921	0.229	-7.892	<0.001
	VS (NV)*TL (MF) — VS(NV)*TL (DP) = 0	-1.119	0.524	-6.762	<0.001
	VS (V)*TL (C) — VS(V)*TL (F) = 0	-0.053	0.012	-0.920	0.001
	VS (V)*TL (C) — VS(V)*TL (MF) = 0	-0.688	0.123	-5.012	<0.001
	VS (V)*TL (F) — VS(V)*TL (MF) = 0	-0.138	0.076	-3.287	0.002
	VS (NV)*TL (C) — VS(NV)*TL (F) = 0	0.198	0.025	1.083	0.035
**TLR4**	VS (NV)*TL (C) — VS(NV)*TL (MF) = 0	0.102	0.076	2.065	0.017
	VS (NV)*TL (F) — VS(NV)*TL (MF) = 0	0.092	0.013	0.533	1.000
	VS (NV)*TL (C) — VS(NV)*TL (DP) = 0	2.341	0.231	8.342	<0.001
	VS (NV)*TL (F) — VS(NV)*TL (DP) = 0	0.833	0.321	5.421	<0.001
	VS (NV)*TL (MF) — VS(NV)*TL (DP) = 0	0.112	0.084	1.098	<0.001
	VS (V)*TL (F) — VS(V)*TL (MF) = 0	0.143	0.092	1.096	<0.001
**TLR9**	VS (NV)*TL (F) — VS(NV)*TL (MF) = 0	-0.045	0.009	-0.120	0.872
	VS (NV)*TL (F) — VS(NV)*TL (DP) = 0	0.341	0.348	2.109	<0.001
	VS (NV)*TL (MF) — VS(NV)*TL (DP) = 0	0.129	0.034	1.073	<0.001
	VS (V)*TL (C) — VS(V)*TL (F) = 0	1.050	0.467	8.666	<0.001
	VS (V)*TL (C) — VS(V)*TL (MF) = 0	2.994	0.539	10.557	<0.001
	VS (V)*TL (F) — VS(V)*TL (MF) = 0	-1.944	0.541	-9.911	<0.001
	VS (NV)*TL (C) — VS(NV)*TL (F) = 0	0.244	0.539	3.544	0.018
**IFN-y**	VS (NV)*TL (C) — VS(NV)*TL (MF) = 0	1.013	0.558	8.998	<0.001
	VS (NV)*TL (F) — VS(NV)*TL (MF) = 0	-2.288	0.492	-10.653	<0.001
	VS (NV)*TL (C) — VS(NV)*TL (DP) = 0	3.129	0.660	12.743	<0.001
	VS (NV)*TL (F) — VS(NV)*TL (DP) = 0	2.885	0.666	11.372	0.094
	VS (NV)*TL (MF) — VS(NV)*TL (DP) = 0	0.597	0.629	5.866	>1.000
	VS (V)*TL (F) — VS(V)*TL (MF) = 0	0.453	0.126	5.183	<0.001
**iNOS**	VS (NV)*TL (F) — VS(NV)*TL (MF) = 0	0.243	0.098	2.087	1.000
	VS (NV)*TL (F) — VS(NV)*TL (DP) = 0	0.112	0.056	2.054	0.982
	VS (NV)*TL (MF) — VS(NV)*TL (DP) = 0	0.023	0.002	0.276	0.162
	VS (V)*TL (F) — VS(V)*TL (MF) = 0	-0.746	0.198	-5.231	<0.001
**CD204**	VS (NV)*TL (F) — VS(NV)*TL (DP) = 0	0.234	0.099	1.076	0.517
	VS (NV)*TL (MF) — VS(NV)*TL (DP) = 0	0.109	0.032	1.043	0.643

SE, standard error., V, vaccinated., NV, non-vaccinated., C, control., F, Focal., MF, multifocal., DP, diffuse paucibacillary., * interaction

Table S4. Results of the post-hoc Tukey's Honestly Significant Difference test for all pairwise comparisons in the model classified with ΔAICC < 2 for the number of TLR1, TLR2, TLR4, IFN-y immunolabeled cells in the scapular lymph node according to the infection (IS) and vaccination status (VS).

**Marker**	**Linear hypotheses**	**Estimate**	**SE**	**t-ratio**	***p* value**
	VS (V) — VS (NV) = 0	0.170	0.051	3.633	0.417
**TLR1**	IS (IF)*VS (V) — IS (IF)*VS (NV) = 0	0.043	0.012	1.762	0.713
	IS (NIF)*VS (V) — IS (NIF)*VS (NV) = 0	0.082	0.034	1.992	0.263
	VS (V) — VS (NV) = 0	0.145	0.029	2.234	0.005
**TLR2**	IS (IF)*VS (V) — IS (IF)*VS (NV) = 0	0.341	0.087	5.811	0.167
	IS (NIF)*VS (V) — IS (NIF)*VS (NV) = 0	0.231	0.023	4.022	0.944
	VS (V) — VS (NV) = 0	-0.130	0.0237	-1.341	0.047
**TLR4**	IS (IF)*VS (V) — IS (IF)*VS (NV) = 0	0.141	0.034	2.235	0.528
	IS (NIF)*VS (V) — IS (NIF)*VS (NV) = 0	0.183	0.054		0.948
	VS (V) — VS (NV) = 0	-0.283	0.031	-1.221	<0.001
**IFN-y**	IS (IF)*VS (V) — IS (IF)*VS (NV) = 0	-0.208	0.010	-2.542	<0.001
	IS (NIF)*VS (V) — IS (NIF)*VS (NV) = 0	-0.095	0.012	-1.435	0.019

SE, standard error., IF, infected)., NIF, non-infected., V, vaccinated., NV, non-vaccinated.,* interaction.

Table S5. Results of the post-hoc Tukey's Honestly Significant Difference test for all pairwise comparisons in the model classified with ΔAICC < 2 for the number of TLR1, TLR2, TLR4, and IFN-y immunolabeled cells in the scapular lymph node according to the type of lesion (TL) present in the intestine and vaccination status (VS).

Marker	Linear hypotheses	Estimate	SE	t-ratio	*p* value
	VS (V)*TL (C) — VS(V)*TL (F) = 0	0.123	0.032	2.654	0.856
	VS (V)*TL (C) — VS(V)*TL (MF) = 0	-0.423	0.127	-7.762	0.003
	VS (V)*TL (F) — VS(V)*TL (MF) = 0	-0.198	0.065	-4.728	0.023
	VS (NV)*TL (C) — VS(NV)*TL (F) = 0	0.121	0.098	2.982	0.982
**TLR1**	VS (NV)*TL (C) — VS(NV)*TL (MF) = 0	0.103	0.054	1.982	1.000
	VS (NV)*TL (F) — VS(NV)*TL (MF) = 0	0.098	0.043	0.986	1.000
	VS (NV)*TL (C) — VS(NV)*TL (DP) = 0	-0.321	0.054	-5.976	<0.001
	VS (NV)*TL (F) — VS(NV)*TL (DP) = 0	-0.301	0.012	-3.754	<0.001
	VS (NV)*TL (MF) — VS(NV)*TL (DP) = 0	-0.124	0.031	-2.763	<0.001
	VS (V)*TL (C) — VS(V)*TL (F) = 0	0.091	0.032	0.922	1.000
	VS (V)*TL (C) — VS(V)*TL (MF) = 0	-0.211	0.021	-3.872	0.002
	VS (V)*TL (F) — VS(V)*TL (MF) = 0	-0.087	0.011	-0.872	0.017
**TLR2**	VS (NV)*TL (C) — VS(NV)*TL (F) = 0	-0.076	0.021	-0.987	1.000
	VS (NV)*TL (C) — VS(NV)*TL (MF) = 0	-0.101	0.023	-1.867	1.000
	VS (NV)*TL (F) — VS(NV)*TL (MF) = 0	-0.192	0.043	-2.666	1.000
	VS (NV)*TL (C) — VS(NV)*TL (DP) = 0	-0.421	0.065	-6.872	0.009
	VS (NV)*TL (F) — VS(NV)*TL (DP) = 0	-0.123	0.021	-2.347	0.321
	VS (NV)*TL (MF) — VS(NV)*TL (DP) = 0	-0.101	0.034	-1.298	0.067
	VS (V)*TL (C) — VS(V)*TL (F) = 0	0.045	0.032	0.972	0.742
	VS (V)*TL (C) — VS(V)*TL (MF) = 0	0.123	0.012	1.089	0.872
	VS (V)*TL (F) — VS(V)*TL (MF) = 0	0.093	0.014	0.765	1.000
	VS (NV)*TL (C) — VS(NV)*TL (F) = 0	-0.087	0.032	-0.411	1.000
**TLR4**	VS (NV)*TL (C) — VS(NV)*TL (MF) = 0	-0.154	0.086	-1.875	1.000
	VS (NV)*TL (F) — VS(NV)*TL (MF) = 0	-0.102	0.034	-1.021	0.209
	VS (NV)*TL (C) — VS(NV)*TL (DP) = 0	-0.322	0.034	-5.421	0.045
	VS (NV)*TL (F) — VS(NV)*TL (DP) = 0	-0.233	0.037	-4.211	0.032
	VS (NV)*TL (MF) — VS(NV)*TL (DP) = 0	-0.123	0.012	-1.423	0.013
	VS (V)*TL (C) — VS(V)*TL (F) = 0	-0.221	0.031	-3.873	0.013
	VS (V)*TL (C) — VS(V)*TL (MF) = 0	-0.312	0.021	-4.912	0.021
	VS (V)*TL (F) — VS(V)*TL (MF) = 0	-0.122	0.065	-2.652	0.098
	VS (NV)*TL (C) — VS(NV)*TL (F) = 0	-0.197	0.023	-2.982	0.001
**IFN-y**	VS (NV)*TL (C) — VS(NV)*TL (MF) = 0	0.023			0.197
	VS (NV)*TL (F) — VS(NV)*TL (MF) = 0	0.122	0.034	1.827	<0.001
	VS (NV)*TL (C) — VS(NV)*TL (DP) = 0	-0.042	0.021	-0.342	0.982
	VS (NV)*TL (F) — VS(NV)*TL (DP) = 0	0.102	0.043	2.867	0.001
	VS (NV)*TL (MF) — VS(NV)*TL (DP) = 0	-0.122	0.022	-2.982	0.013

SE, standard error., V, vaccinated., NV, non-vaccinated., C, control., F, Focal., MF, multifocal., DP, diffuse paucibacillary., * interaction
